# UNVEILING THE DIFFERENTIAL IMPACT OF NEGATIVE AND POSITIVE AFFECT ON SUBJECTIVE COGNITIVE PERCEPTIONS

**DOI:** 10.1093/geroni/igae098.0024

**Published:** 2024-12-31

**Authors:** Jacklyn Gehling, Hannah Marx, Ravneet Dhaliwal, Steven Harris, Frank Robertson, Claudia Jacova Chenoweth

**Affiliations:** Pacific University, Hillsboro, Oregon, United States; Pacific University, Hillsboro, Oregon, United States; Pacific University, Hillsboro, Oregon, United States; Pacific University, Hillsboro, Oregon, United States; Pacific University, Hillsboro, Oregon, United States; Pacific University, Hillsboro, Oregon, United States

## Abstract

Subjective Cognitive Decline (SCD) is a state of perceived decline in cognitive abilities that may mark Alzheimer’s disease onset and may be influenced by affective and personality factors. This study aimed to examine the distinct impact of negative and positive affect on SCD burden in older adults. The sample included 279 cognitively healthy, community-dwelling participants, aged 60-96 (M=73.8), 52% female, education 10-20 years (M=14.9). Participants completed an online survey including demographics, depression (PHQ-2, M=.51), perceived memory difficulties (ECog-Memory, M=12.4), and affect (Positive and Negative Affect Schedule, PANAS, M=13.7 and M=7.1 respectively). Separate regression analyses of ECog-Memory showed that PANAS-Negative (B=.35, ΔR²=.05, p<.001) but not PANAS-Positive (B=-.08, ΔR²=.008, p=.12), added explained variance beyond demographics and depression. Further exploration of PANAS-Positive, incorporating its interaction with education due to trending significance (p=.08), showed that PANAS-Negative (B=.35, p<.001), but not PANAS-Positive (B=.52) or the interaction (B=-.04), contributed explained variance (ΔR²=.07). Our findings highlight the significant influence of negative affect on the subjective cognitive perceptions of older adults. Elevated negative affect is correlated with diminished subjective cognitive perceptions, and notably, this association remains independent of endorsed depression. Interestingly, positive affect, a known protective factor in AD-related cognitive decline, does not seem to have an impact on subjective cognition.

